# Benefitting from Dopant Loss and Ostwald Ripening in Mn Doping of II-VI Semiconductor Nanocrystals

**DOI:** 10.1186/s11671-015-1123-9

**Published:** 2015-10-28

**Authors:** You Zhai, Moonsub Shim

**Affiliations:** Department of Materials Science and Engineering, University of Illinois at Urbana-Champaign, Urbana, IL 61801 USA

**Keywords:** Ostwald ripening, Dopant loss, Beneficial effect, Doping semiconductor nanocrystals, Size dependence, Surface cation exchange, Dopant clustering

## Abstract

Annealing or growth at high temperatures for an extended period of time is considered detrimental for most synthetic strategies for high-quality Mn-doped II-VI semiconductor nanocrystals. It can lead to the broadening of size distribution and, more importantly, to the loss of dopants. Here, we examine how ripening can be beneficial to doping in a simple “heat-up” approach, where high dopant concentrations can be achieved. We discuss the interplay of the loss of dopants, Ostwald ripening, and the clustering of Mn near the surface during nanocrystal growth. Smaller nanocrystals in a reaction batch, on average, exhibit higher undesirable band-edge photoluminescence (PL) and lower desirable dopant PL. The optimization of dopant loss and the removal of such smaller undesirable nanocrystals through Ostwald ripening along with surface exchange/passivation to remove Mn clustering lead to high Mn PL quantum yields (45 to 55 %) for ZnS_x_Se_1−x_, ZnS, CdS, and CdS_x_Se_1−x_ host nanocrystals. These results provide an improved understanding of the doping process in a simple and potentially scalable synthetic strategy for achieving “pure” and bright dopant emission.

**Electronic supplementary material:**

The online version of this article (doi:10.1186/s11671-015-1123-9) contains supplementary material, which is available to authorized users.

## Background

Doping semiconductors with transition metals can impart new and useful optical, electronic, and/or magnetic properties [1]. Additional prospects arising from combining quantum size effect with dopant-enabled capabilities have led to the exploration of various dopants in several different types of semiconductor nanocrystals (NCs) [1–10], with Mn-doped II-VI NCs garnering much attention [2, 4, 6–19]. In addition to intriguing magneto-optical properties [20, 21], energy transfer from the photo-excited host to the dopant can lead to high quantum yield (QY) photoluminescence (PL) from relaxed spin-forbidden ^4^T_1_ → ^6^A_1_ transition [22]. The high PL QYs and large Stokes shift between the host absorption edge and Mn PL in Zn-based II-VI NCs are especially promising for developing down-converters for solar cells [23, 24] and solid-state lighting [25] and fluorescent markers for biomedical imaging [17] and sensing [26]. Emission wavelength tunability via lattice strain can provide additional benefits [27, 28]. Hence, Mn-doped Zn-based II-VI NCs are often considered as heavy-metal-free, low-toxicity alternatives to CdSe and related NCs.

While Mn has been relatively easy to dope into II-VI NCs, especially ZnS and CdS, at low temperatures, the resulting NCs have often been of low quality [11–14, 18, 29, 30]. Mn-doped NCs with high crystallinity and good control over size/size distribution can be achieved with the “hot-injection” method [2, 15], but low dopant concentrations (far below the bulk solubility) and the statistical nature of dopant inclusion lead to a significant fraction of NCs being undoped [2, 3, 15, 16]. High temperature growth/ripening often necessary to achieve high-quality NCs is at the root of these problems. At elevated temperatures, “self-purification” may occur via dopant diffusion and eventual ejection out of the lattice [31, 32]. Norris et al. have argued that *intraparticle* ripening, rather than self-purification, may be the cause of dopant loss [16, 33]. In addition, smaller particles tend to be more difficult to dope [16, 18] and there appears to be a critical NC size smaller than which dopants cannot be incorporated and larger than which dopants are incorporated at a constant rate [20, 21, 31, 34]. While many factors contribute and doping mechanisms remain under debate [16, 31], annealing or ripening of NCs is usually considered to be detrimental, e.g., even if dopant adsorption on NC surfaces is favorable and even if the NCs are in the size regime of favorable dopant incorporation rate, ripening [33] and/or dopant diffusion during growth [8, 9, 31, 32] can lead to the loss of dopants. In fact, Norris et al. have shown that maintaining precursor concentrations during growth can minimize intraparticle ripening-induced dopant loss [33].

Other promising approaches to Mn doping separate dopant incorporation from nucleation and/or growth of the host NCs [4, 6, 7, 9, 27, 35, 36]. “Nucleation doping” is a particularly successful example where extremely small Mn chalcogenide core is formed first and the host II-VI NC is grown as the shell [4, 6, 7, 9, 17]. However, a relatively low shell growth temperature is required to prevent separate nucleation/growth of undoped NCs [4] and Mn diffusion out to the surface [7, 9]. Hence, extended high temperature growth can also be detrimental to the nucleation doping approach. Nevertheless, optimization efforts have led to high-quality materials that can be stable at relatively high growth temperatures [6].

Cation exchange, where high-quality host NCs can be grown separately, is a potential approach that can avoid ripening of the host material altogether but has been limited to low-temperature Mn doping of magic-sized clusters of ZnTe [37] presumably due to high temperatures necessary to drive incorporation of Mn^2+^ which leads again to the ripening problem for most II-VI NCs [38, 39]. Alternatively, Gamelin et al. have introduced a diffusion doping approach successfully incorporating Mn in difficult-to-dope CdSe NCs and even in CdSe nanorods [39]. The key innovation of diffusion doping is controlling the solution and crystal lattice chemical potentials to incorporate both dopants and anions from solution to the existing NCs without significant host cation loss and therefore avoiding ripening.

In most Mn doping approaches developed to date, growth or annealing at high temperatures for an extended period of time is considered to be an unfavorable process that should be avoided or minimized as much as possible. It can lead not only to the broadening of size distribution but also to the detrimental loss of dopants. However, high temperatures are usually necessary to achieve high crystallinity and high PL QYs, and therefore, Mn loss and ripening are usually unavoidable. Here, we examine a simple and potentially scalable “heat-up” synthetic strategy where all precursors for II-VI host material and the Mn dopant are added together at a low temperature and heated to a high growth temperature. Optimizing the time of growth at a high temperature can lead to high Mn PL QY while suppressing the band-edge PL. Mn PL QYs of ~50 % are demonstrated for several different II-VI host materials. With initial high Mn concentrations and the smaller NCs having higher band-edge PL and lower Mn PL, both dopant loss and Ostwald ripening have beneficial effects in achieving desired PL properties.

## Methods

### Chemicals

Zinc stearate (ZnSt_2_, technical grade), selenourea (98 %), manganese chloride (MnCl_2_, anhydrous beads, 99.999 %), zinc acetate (Zn(OAc)_2_, 99.99 %), cadmium acetate dihydrate (Cd(OAc)_2_·2H_2_O, 98 %), manganese acetate tetrahydrate (Mn(OAc)_2_·4H_2_O, 99.99 %), sulfur powder (S, 98 %), 1-dodecanethiol (DDT, ≥ 98 %), oleylamine (OAm, technical grade), and coumarin 153 (C153, dye content 99 %) were purchased from Sigma-Aldrich and used without further purification. All syntheses were carried out using the standard Schlenk technique.

### Synthesis of Mn-doped ZnS_x_Se_1−x_ NCs

A Se precursor solution was first prepared by dissolving 0.5 mmol of selenourea [40] in 5 mL of OAm at 200 °C under N_2_ after degassing under vacuum at 100 °C for 30 min, and stored at room temperature (RT) under N_2_. In a separate flask, 0.2 mmol of ZnSt_2_ and 0.04 mmol of MnCl_2_ were dissolved in 0.25 mL of DDT and 8 mL of OAm at 120 °C under N_2_ after degassing at 100 °C for 30 min. To this reaction mixture, 2 mL of selenourea solution was added via a syringe, and the temperature was raised to 260 °C in ~10 min. The reaction mixture was kept at 260 °C for various times as indicated before the heating mantle was removed. Once the reaction mixture was cooled to RT, a surface exchange/passivation step was carried out by adding 0.5 mmol of ZnSt_2_ to the reaction mixture and raising the temperature up to 250 °C in ~10 min. The reaction mixture was kept at 250 °C for 2 h before the heating mantle was removed. Small aliquots of the reaction mixture were taken throughout the reaction and precipitated with methanol and redissolved in chloroform for optical measurements. Additional two cycles of methanol/chloroform purification were carried out before structural and compositional analyses. This purification procedure was the same for all syntheses described below.

To support the effect of Ostwald ripening, size-selective precipitation was carried out by adding methanol dropwise to the reaction mixture at RT until slight cloudiness persisted. The precipitate was separated from the supernatant by centrifugation at 3500 rpm for 5 min. Then the precipitate was redissolved in chloroform for optical measurement whereas more methanol was added dropwise to the supernatant for further size selection. This procedure was repeated several times until there was insufficient amount of precipitate for optical characterization.

### Synthesis of Mn-doped ZnS NCs

A solution of S was first prepared by dissolving 0.25 mmol of S powder in 5 mL of OAm at 100 °C under N_2_ after degassing at 100 °C for 30 min, and stored at RT under N_2_. In a separate flask, 0.2 mmol of Zn(OAc)_2_ and 0.04 mmol of Mn(OAc)_2_·4H_2_O were dissolved in 0.25 mL of DDT and 8 mL of OAm at 160 °C under N_2_ after degassing at 100 °C for 30 min. S solution (2 mL) was then added to the Zn(OAc)_2_/Mn(OAc)_2_ solution, and the temperature was raised to 250 °C in ~8 min. The reaction mixture was kept at 250 °C for various times before the heating mantle was removed. At RT, 1:1 volume ratio of methanol was added to precipitate the NCs from the reaction mixture. Another redissolution/precipitation was carried out with chloroform and methanol, and the precipitate was redissolved in chloroform. Surface exchange/passivation was then carried out by first dissolving 0.5 mmol of ZnSt_2_ in 8 mL of OAm after degassing at 100 °C for 30 min. Then, the solution of Mn-doped NCs was added and the chloroform was evaporated under vacuum. The temperature was raised to 230 °C in ~9 min and maintained for 30 min before the heating mantle was removed.

We note the similarity of this synthesis to Mn-doped ZnS nanorods [41] with the exception of the use of ZnSt_2_ instead of Zn(NO_3_)_2_ as the zinc precursor. We also examined other Zn precursors, Zn(OAc)_2_, ZnCl_2_, and Zn(acetylacetonate)_2_, and observed that these precursors lead to spherical dots (while ZnSO_4_ leads to a mixture of dots and nanorods), suggesting that dots may be the more general case.

### Synthesis of Mn-doped CdS NCs

A S precursor solution was first prepared by dissolving 0.2 mmol of S powder in 3 mL of OAm at 100 °C under N_2_ after degassing at 100 °C for 30 min, and stored at RT under N_2_. In a separate flask, 0.2 mmol of Cd(OAc)_2_·2H_2_O and 0.04 mmol of Mn(OAc)_2_·4H_2_O were dissolved in 0.25 mL of DDT and 8 mL of OAm at 160 °C under N_2_ after degassing at 100 °C for 30 min. S solution (1 mL) was then added into the Cd(OAc)_2_/Mn(OAc)_2_ solution, and the temperature was raised to 250 °C in ~6 min. The reaction mixture was kept at 250 °C for various times before the heating mantle was removed. Batch purification and surface exchange/passivation were the same as described above for Mn-doped ZnS NCs.

### Synthesis of Mn-doped CdS_x_Se_1−x_ NCs

Selenourea (0.2 mmol) was first dissolved in 3 mL of OAm at 200 °C under N_2_ after degassing at 100 °C for 30 min, and stored at RT under N_2_. In a separate flask, 0.2 mmol of Cd(OAc)_2_·2H_2_O and 0.04 mmol of Mn(OAc)_2_·4H_2_O were dissolved in 1 mL of DDT and 8 mL of OAm at 120 °C under N_2_ after degassing at 100 °C for 30 min. Selenourea solution (1 mL) was added into Cd(OAc)_2_/Mn(OAc)_2_ solution, and the temperature was raised to 250 °C in ~6 min. The reaction mixture was kept at 250 °C for various times (less than 40 min to avoid the band gap being too small such that the Mn emission is suppressed [42]) before the heating mantle was removed. Batch purification and surface exchange/passivation were the same as described above for Mn-doped ZnS NCs.

### Characterization

Transmission electron microscopy (TEM) was carried out on a JEOL 2100 TEM. Scanning TEM (STEM) images and energy-dispersive X-ray spectroscopy (EDS) were recorded using a JEOL 2010 FETEM with an Oxford EDS detector. Samples were prepared by drop-drying the purified NC solution in chloroform onto a carbon-coated copper grid. The atomic percentages from EDS data were averaged over more than five individual areas and each area contained at least 100 NCs. Due to the low contrast in bright-field TEM images (especially for small NCs in early stages), the diameters of the samples were measured from the STEM images perpendicular to the scanning direction in case of scanning noise, and averaged over above 100 NCs. Powder X-ray diffraction (XRD) patterns were recorded with Siemens-Bruker D5000 diffractometer. Samples were prepared by drop-casting a concentrated NC solution in chloroform on a low background quartz substrate. UV-vis absorption and PL spectra were recorded at RT with Agilent 8453 diode array UV-vis spectrophotometer and Horiba Jobin-Yvon Fluoromax-3 spectrofluorometer, respectively. Unless otherwise noted, the excitation wavelengths for measuring Mn PL QY after surface exchange/passivation were 380 nm for Mn-doped ZnS_x_Se_1−x_ NCs, 290 nm for Mn-doped ZnS NCs, 420 nm for Mn-doped CdS NCs, and 430 nm for Mn-doped CdS_x_Se_1−x_ NCs. A 500-nm longpass color filter was used for Mn-doped ZnS NCs. C153 (QY = 54.4 % in ethanol [43]) was used as the reference dye for Mn PL QY measurements due to its large Stokes shift and similar absorption/PL spectral range with the doped NCs (Additional file 1: Figure S1). No reference dye was selected to measure the relative QY of the band-edge PL for Mn-doped ZnS_x_Se_1−x_ NCs. Therefore, only the integrated PL peak areas normalized to the absorbance at the excitation wavelength were compared. For the Mn-doped ZnS NCs, due to the absorption in the UV range, the relative QY versus C153 was estimated using the emission spectrum of the Xe lamp in the spectrofluorometer. EPR measurements were performed on a concentrated chloroform suspension of NCs at 9.20 GHz using a Varian E-line X-band spectrometer at RT.

## Results and Discussion

While there are currently several different approaches to synthesizing Mn-doped II-VI semiconductor NCs, we focus here on the heat-up synthesis because high-quality/high-PL materials can be achieved with simplicity and because of its potential for scale up and extension to anisotropic structures [41, 44, 45]. Furthermore, since all reagents are mixed together initially, growth/ripening and Mn incorporation/loss may be more difficult to separate and control than other synthetic methods. Therefore, an understanding of how dopant incorporation and loss are affected by high temperature growth/annealing is of critical importance in order to take advantage of potential benefits of the heat-up synthesis.

Once Mn impurities are incorporated into the NCs, high growth/annealing temperatures can cause undesirable decrease in the dopant concentration and increase the number of undoped NCs, leading to a decrease in the Mn PL and an increase in the band-edge PL [32, 46]. However, in the high dopant concentration regime (i.e., significantly larger than one dopant per particle), reducing the number of dopant atoms per NC might be desirable [19, 47, 48]. In this sense, the heat-up synthesis where Mn dopants may be incorporated at high concentrations (initially at low temperatures) presents a potential system where Mn dopant loss may be beneficial. Hence, we focus on the doping process in the heat-up synthesis and consider both growth via Ostwald ripening and dopant loss during NC growth/annealing. We note that annealing a purified solution of NCs in the absence of potential reaction byproducts might appear to better serve in illustrating Ostwald ripening effects on doping. However, the effects of changing precursor concentrations on the overall chemical equilibrium between the solution and the nanocrystalline phases [39] complicate the situation. Hence, we limit our discussion here to Ostwald ripening in the growth solution. We also note that our main goals here are to identify trends arising in the evolution of spectral features during the heat-up synthesis and to provide a better understanding of how these trends are related to the doping/growth processes occurring. We first discuss ZnS_x_Se_1−x_ as the host NC since much less work has been reported on alloyed materials [10, 49] and alloying can provide tunability of absorption across a broader spectral range which would be beneficial in applications such as down-converters. The generality of our findings are demonstrated by extending our studies to other host materials (i.e., ZnS, CdS, and CdS_x_Se_1−x_).

### Structural and Spectral Features of Mn-doped ZnS_x_Se_1−x_ NCs

**Fig. 1:**
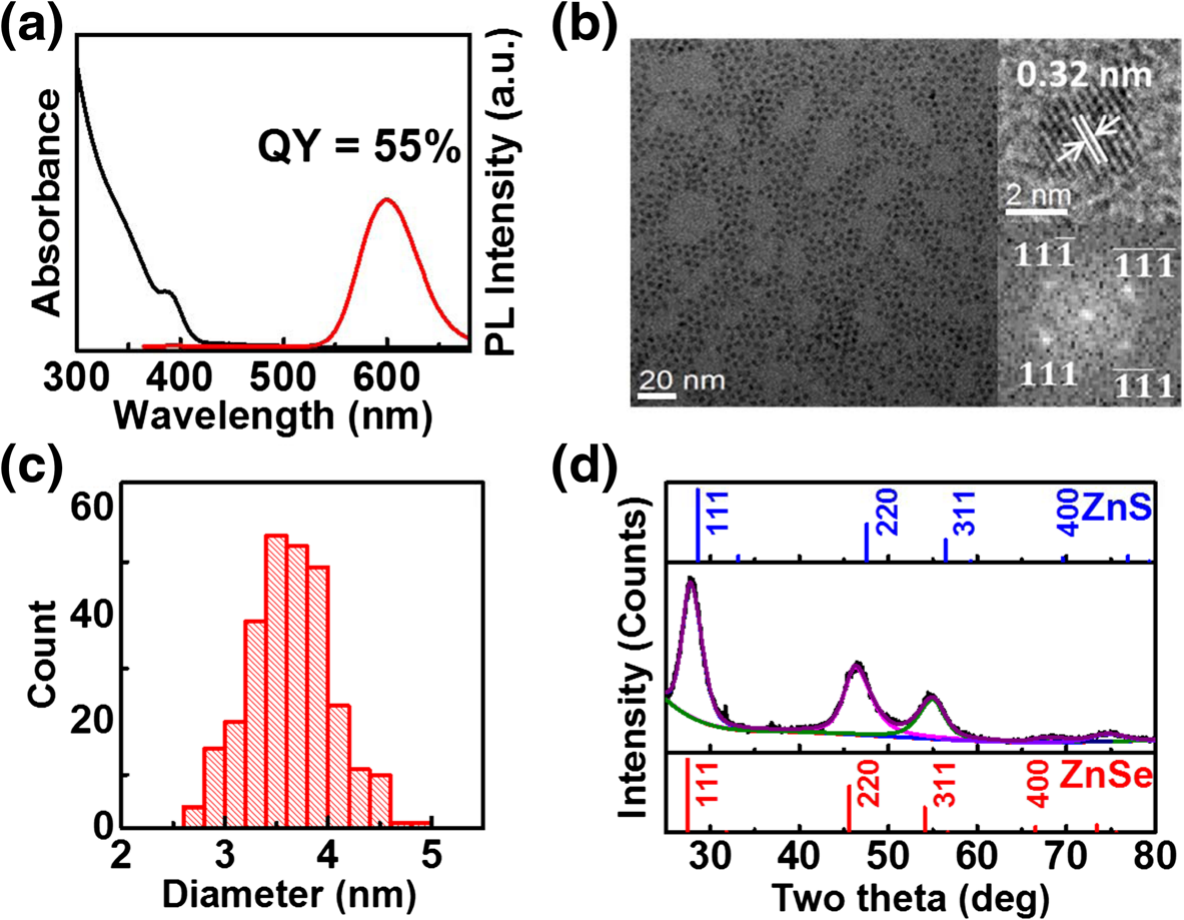
Absorption/PL spectra (**a**), TEM image along with HRTEM image and corresponding FFT pattern (**b**), diameter distribution (**c**), and powder XRD (ICDD # 04-001-6858 for ZnSe and ICDD # 04-001-6857 for ZnS also shown) (**d**) of Mn-doped ZnS_x_Se_1−x_ NCs after surface exchange/passivation

Figure 1 shows the absorption/PL spectra, TEM images, diameter histogram, and XRD results of Mn-doped ZnS_x_Se_1−x_ NCs. Precursors for Zn, Mn, S, Se, and capping ligands are mixed together at 120 °C, heated to 260 °C, and annealed/grown for 90 min for the case shown in Fig. 1. Although the initial PL QY is low, we observe that the addition of ZnSt_2_ at RT and subsequent annealing at 250 °C for 2 h leads to strong Mn PL with a QY of 55 % with the band-edge PL being essentially completely quenched. The addition of extra zinc precursors has been reported to improve PL QY possibly through the formation of a type I passivating ZnS shell [50]. It may also cause surface cation exchange, which was previously thought unfavorable due to the loss of dopant [46].

The NCs are nearly spherical with a *d*-spacing of 0.32 nm between {111} planes as observed in the high-resolution TEM (HRTEM) image (Fig. 1b). XRD results reveal zinc-blende structure (Fig. 1d). The lattice constant of 0.554 nm is consistent with that expected from the *d*-spacing measured by HRTEM and lies between ZnSe (0.567 nm) and ZnS (0.541 nm), implying an alloyed ZnS_x_Se_1−x_ composition or possibly a gradient composition. Given that DDT (the S source) should react at a higher temperature (above 230 °C) [51] than selenourea (~160 °C) [38], we expect our resulting NCs to be closer to the latter case of gradient composition with ZnSe-rich core and ZnS-rich outer layer. As discussed below, EDS analysis supports this expectation. Prior to the surface exchange/passivation with additional ZnSt_2_, the lattice constant is slightly larger at 0.560 nm, indicating that this step further enriches ZnS composition near the surface. The diameter increases only by ~0.5 nm upon surface exchange/passivation with additional ZnSt_2_, suggesting about a monolayer of ZnS growth.

**Fig. 2:**
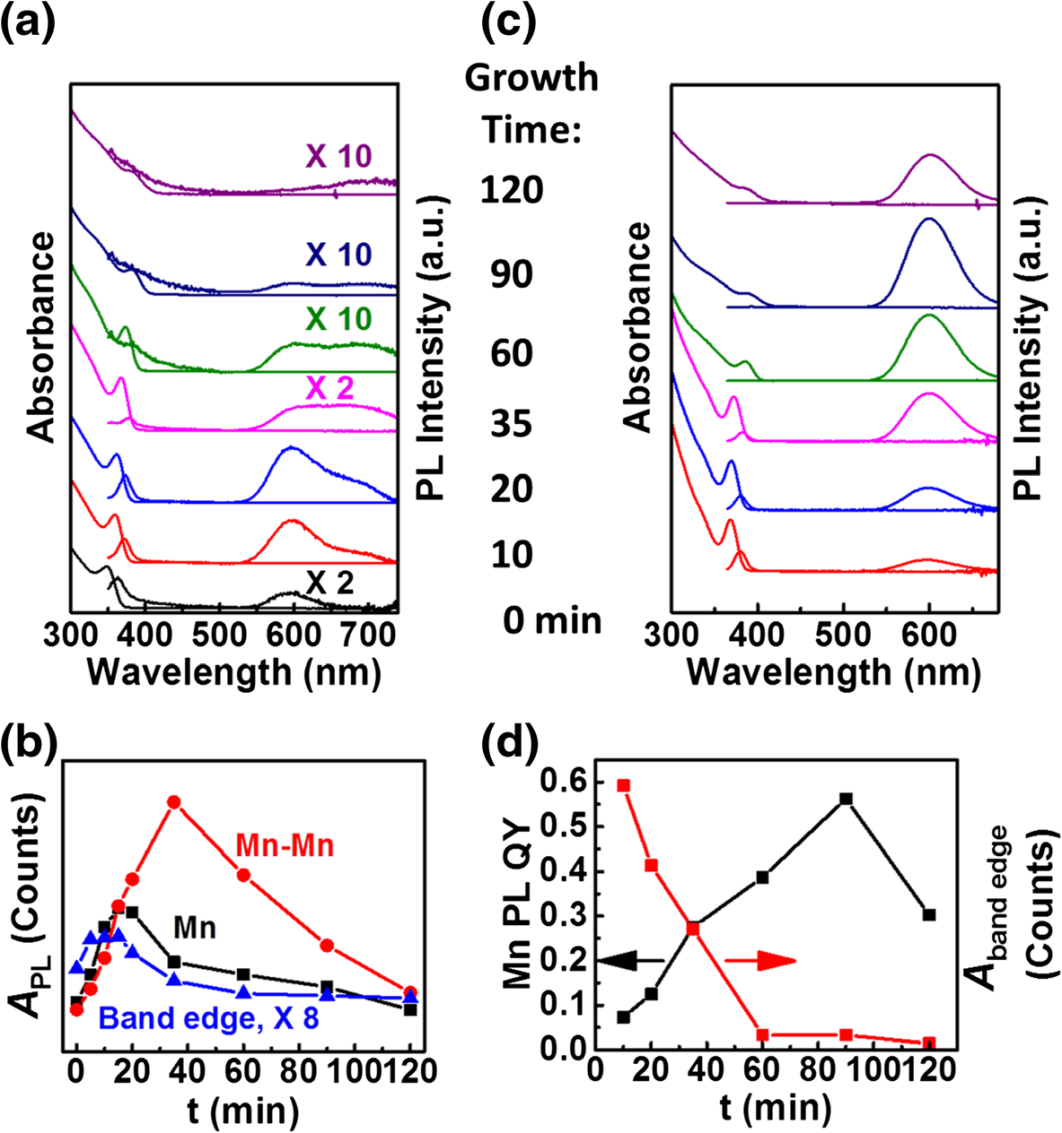
**a** Absorption/PL spectra of Mn-doped ZnS_x_Se_1−x_ NCs at the indicated growth times before surface exchange/passivation. The excitation wavelength was 350 nm for the Mn PL due to the initial shorter absorption wavelengths (measured with a 500-nm longpass filter) and 330 nm for band-edge PL. Background rise in the UV region is due to organic species in the solution. **b** Integrated PL areas of band-edge, isolated Mn, and Mn-Mn emissions for NCs with different growth times before surface exchange/passivation. **c** Absorption/PL spectra of Mn-doped ZnS_x_Se_1−x_ NCs after surface exchange/passivation, which took place after the indicated growth times. The full PL spectra were collected with a 350 nm excitation wavelength. **d** Growth-time dependence of Mn PL QYs and integrated band-edge PL areas for NCs after surface exchange/passivation. Spectra in **a** and **c** are offset for clarity. All PL spectra are normalized to the absorbance at the excitation wavelength

Figure 2a shows how the absorption and PL spectra evolve with growth time. All PL spectra here and throughout are normalized to the absorbance at the excitation wavelength. The times indicated here and throughout refer to the reaction time after the growth temperature has been reached. Even at *t* = 0 min, there are already three distinct features in the PL spectrum: band-edge emission at ~360 nm, Mn emission at ~590 nm, and a broader feature at ~680 nm. Based on previous reports [6, 12, 13, 29, 30, 52] and correlation to EPR and EDS results discussed below, we attribute the long wavelength feature to emission associated with Mn-Mn interaction arising from local clustering of Mn. We can rule out isolated surface Mn and deep-trap emission since distorted ligand/crystal field at the surface causes isolated Mn PL peak wavelength shift to only ~620 nm [6, 53], and the same synthesis without Mn leads to mainly band-edge emission with a weak deep-trap emission between 450 and 550 nm when observable (Additional file 1: Figure S2), respectively. Continued growth at 260 °C leads to an increasing then decreasing PL intensity for all three features (Fig. 2b). However, when the maximum intensity is reached for each PL peak is different. The maximum intensity for the band-edge emission is reached first at ~10 min, followed by the isolated Mn PL at ~15 min, and then the Mn-Mn interaction PL at ~35 min. The same trends are seen for different initial Mn precursor amounts, but the maxima shift to earlier or later times for higher or lower amounts, respectively (Additional file 1: Figure S3).

Figure 2c and d show how the absorption and PL spectra change upon surface exchange/passivation with additional ZnSt_2_ after the indicated growth times. The narrow band-edge absorption peak is well maintained with a slight red-shift, and essentially, a complete loss of Mn-Mn interaction PL is seen upon ZnSt_2_ treatment for all growth times. This loss is accompanied by a striking increase of 2 to 4 orders of magnitude in the main Mn PL peak at ~590 nm. As shown in Fig. 2d, the isolated Mn PL QY increases to 55 % with increasing growth time up to ~90 min for the particular Mn precursor concentration shown. The intensity of the band-edge emission, however, shows the opposite trend of *decreasing* with longer growth time for both before (*t* > ~10 min) and after ZnSt_2_ treatment. This observation may be surprising (as discussed further later) since Mn concentration is decreasing with growth time, and therefore, one might expect more NCs that emit at the band edge to be appearing.

### Composition and Mn Distribution Evolution During Growth and Surface Exchange/Passivation

**Fig. 3:**
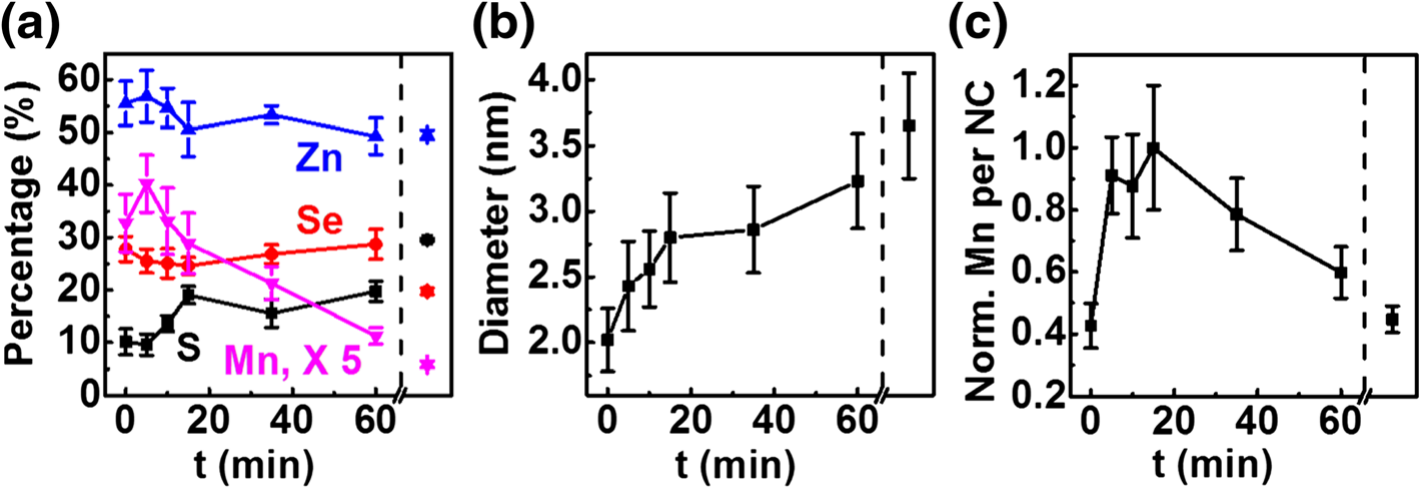
Growth-time dependence of atomic percentages of Zn, Se, S, and Mn (**a**), average diameter (**b**), and average number of Mn per NC (normalized to set the maximum as 1) (**c**) of Mn-doped ZnS_x_Se_1−x_ NCs before (on the *left side* of the *dashed line*) and after (on the *right side* of the *dashed line*) surface exchange/passivation

EDS, size, and EPR analyses provide insights on how Mn concentration evolves over time. The samples for EDS and EPR measurements were purified thoroughly to remove loosely bound Mn. Composition (corresponding EDS spectra are shown in Additional file 1: Figure S4) and size evolution during growth are shown in Fig. 3a and b, respectively. The strong signal from Mn in the EDS spectra for our heavily doped NCs makes this composition analysis more reliable [8, 11]. There is a fast diameter increase for the first 15 min followed by a slower growth. The initial fast increase may be attributed to growth mainly from direct incorporation/reaction of the precursors, whereas the later slower growth may arise from Ostwald ripening after the initial precursors have depleted. EDS results show that the relative Zn and Se amounts do not change very much throughout the growth. However, both Mn and S compositions change significantly especially during the initial 15 min of fast growth. The increase in S may be understood based on the less reactive DDT being the S source and that it is in excess. This increase in S should lead to a ZnS-rich outer layer which should in turn enhance Mn incorporation [16]. Figure 3c shows how the normalized product of the relative Mn amount from EDS and the average volume of NCs from the diameter analysis, which is indicative of how the average number of Mn dopants per NC, change over time. Initially, Mn increases along with S but once the maximum Mn concentration is reached at ~15 min, ripening takes over and Mn loss from the NCs initiates. Upon surface exchange/passivation with ZnSt_2_, there is a further drop in Mn accompanied by an increase in S and a decrease in Se. This observation may be explained by a portion of Mn dopants being at or near the surface and the ZnSt_2_ treatment in the presence of excess DDT passivating the surface with ZnS shell while, at the same time, replacing some of the surface/near-surface Mn cations [46].

**Fig. 4:**
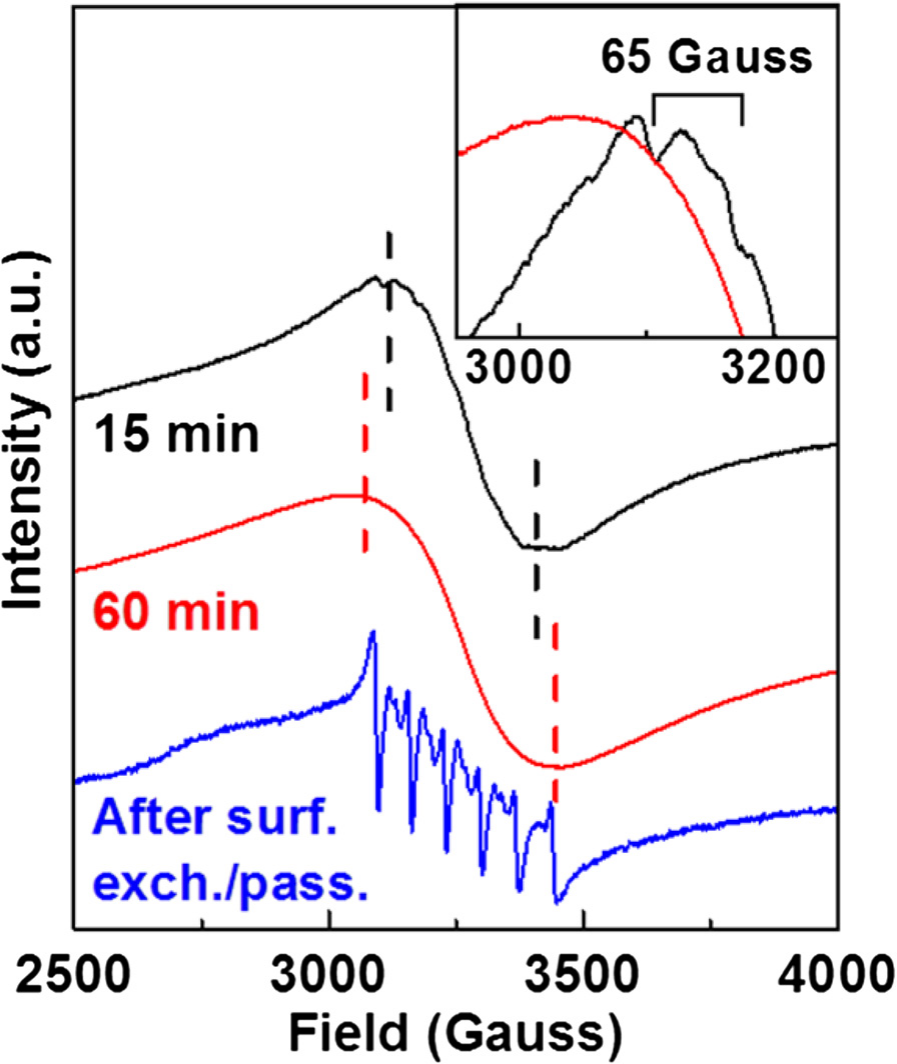
EPR spectra of Mn-doped ZnS_x_Se_1−x_ NCs with short (15 min) and long (60 min) growth times before (the *dashed lines* indicate peak-to-peak position of the broad isotropic resonance) and after surface exchange/passivation. *Inset* magnifies the maximum intensity region for the short and long growth times with the short growth time spectrum showing noticeable hyperfine splitting

EPR measurements (Fig. 4) further help to elucidate how the Mn distribution, or the local concentration, varies during growth and surface exchange/passivation. During an early stage of growth (15 min), there is a broad resonance overlaid with a weak but observable six-line hyperfine splitting (inset). The six-line feature with hyperfine splitting of 63 × 10^−4^ cm^−1^ corresponds to isolated lattice Mn and lies between those of Mn-doped ZnS (64.5 × 10^−4^ cm^−1^) [54] and ZnSe (61.7 × 10^−4^ cm^−1^) [15], consistent with ZnS_x_Se_1−x_ (graded) alloy composition we expect. The broad resonance corresponds to clustering of Mn dopants [11–14, 18, 30] and is also consistent with the long wavelength PL arising from Mn-Mn interaction. We note that such a broad isotropic resonance has rarely been reported for high temperature syntheses because the Mn concentration is usually low for these cases [2, 15]. As growth occurs, only the broad resonance remains and its peak-to-peak width increases (60 min) [11, 14], indicating that clustering of Mn dopants continues (a local Mn concentration increase) despite the overall Mn concentration decrease. This observation is consistent with the continued increase in the Mn-Mn interaction PL past the maximum Mn concentration time. We note that, although the location of Mn was not identified, a similar clustering has been reported for Mn-doped CdSe NCs [55]. Upon surface exchange/passivation using ZnSt_2_, the sharp six-line resonance becomes the salient feature in the EPR spectrum indicating that the majority of the NCs now have isolated Mn centers in the lattice (the disappearance of the broad resonance excludes the EPR signal arising from leftover Mn precursors), consistent with the concurrent large increase in the isolated Mn PL. These observations indicate that the majority of the clustering of Mn occurs at or near the surface of the NCs and are removed upon ZnSt_2_ treatment through cation exchange, which is further supported by the decrease in Mn composition upon surface exchange/passivation as measured by EDS (Fig. 3a).

### Consequences of Ostwald Ripening — Loss of Undesirable NCs

**Scheme 1:**
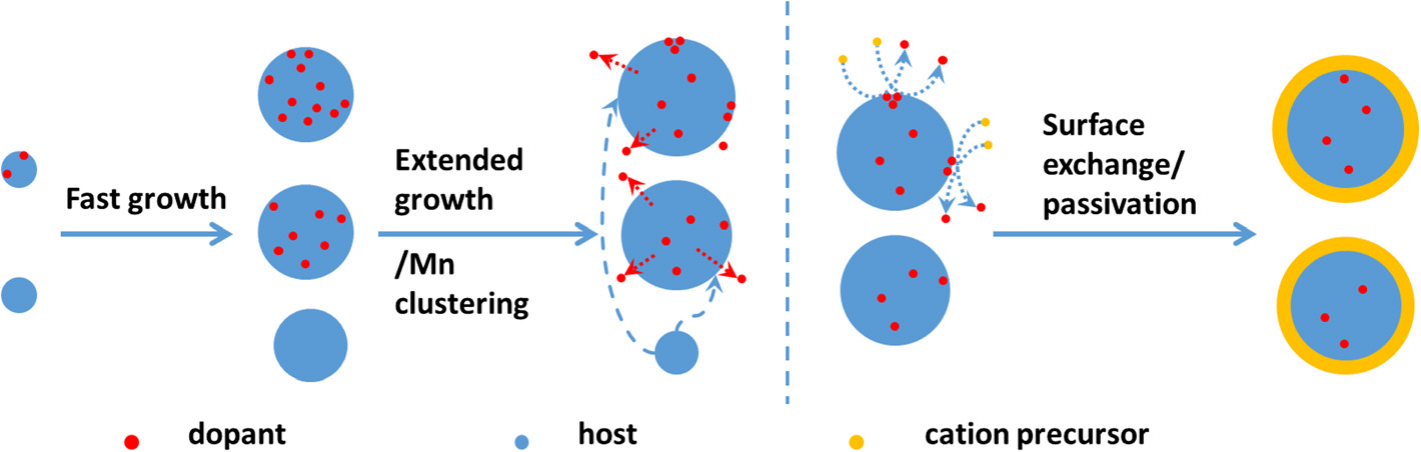
Illustration of the possible doping process involving Mn loss and Ostwald ripening along with Mn clustering near the surface during extended growth, and surface exchange/passivation

Given the three main PL features in Fig. 2, we can consider how the relative populations of the three types of NC species, namely (1) ones with mainly band-edge emission, (2) ones with mainly isolated Mn PL ~590 nm, and (3) ones with mainly Mn-Mn interaction PL ~680 nm, change during growth to better understand the final optical properties. While this classification may be an over simplification of the real system, we believe it helps to elucidate the key elements of growth and doping. Elemental mapping of the individual doped NCs by combining atomic-resolution STEM, electron energy-loss spectroscopy, and simulation [56] may provide direct and more satisfying evidence for the dopant distribution and the doping process. However, the small size and the instability under intense electron beam of our samples as well as the statistical nature of doping [3] make such characterization less reliable and beyond the scope of current study. Nevertheless, based on the above discussions on how absorption, PL, EDS, and EPR change over the reaction time, the emerging picture is that Mn first gets incorporated into the NCs in early stages (e.g., during the heat-up before the final growth temperature is reached), but there is a distribution of all three types of NCs. Once the precursors are depleted, Ostwald ripening and loss of Mn centers occur but there is a concurrent local clustering of the dopants at/near the surface of the NCs. The final ZnSt_2_ treatment leads to the passivation of the surface and, perhaps more importantly, to a significant removal of Mn clustering via cation exchange, ultimately leading to high isolated Mn PL QY. Scheme 1 represents these possible key (simplified) processes occurring in Mn doping via the heat-up synthesis.

An important observation to note is that the highest final Mn PL QY does not correspond to the growth time that gives rise to the highest isolated Mn PL intensity prior to ZnSt_2_ surface exchange/passivation. Rather, the optimum growth time corresponds to the case where the presence of Mn-Mn interaction PL is significant and the band-edge emission is minimized. Hence, the removal of local Mn clustering by surface exchange/passivation is critical in unveiling the role of ripening on Mn doping of NCs here. Notice that the Mn PL QY (after surface exchange/passivation) increases up to a growth time of 90 min (Fig. 2d). This isolated Mn PL increase is accompanied by the band-edge PL *decrease*. This result may be expected of more Mn incorporation leading to a larger number of doped NCs. However, the increase in isolated Mn PL QY and the decrease in band-edge PL continue while the Mn concentration in the NCs is actually *decreasing* (Fig. 3c). Given the high Mn concentration in our case [49], Mn loss may explain the increasing Mn PL (i.e., there may be an optimal doping level above and below which Mn PL is lower) [19, 47, 48], but the concurrent decrease in the band-edge PL is contradictory to what is expected from Mn loss.

An increase in the band-edge PL is expected when dopant loss leads to an increasing number of NCs with mainly band-edge PL. In the high dopant concentration regime, dopant loss can reduce Mn concentration in NCs, but the total number of such NCs can remain the same, leading to a low or quenched band-edge PL that is unchanging. Additional file 1: Figures S2B and C compare changes in the band-edge PL integrated area (normalized to absorbance at the excitation wavelength) for the undoped and doped cases before ZnSt_2_ treatment with the undoped case showing increasing band-edge PL QY with size. Hence, one would expect either increasing or unchanging band-edge PL as Mn loss continues to occur during growth in our situation. In contrast, the *band-edge PL actually decreases* despite decreasing Mn concentration with growth time.

**Fig. 5:**
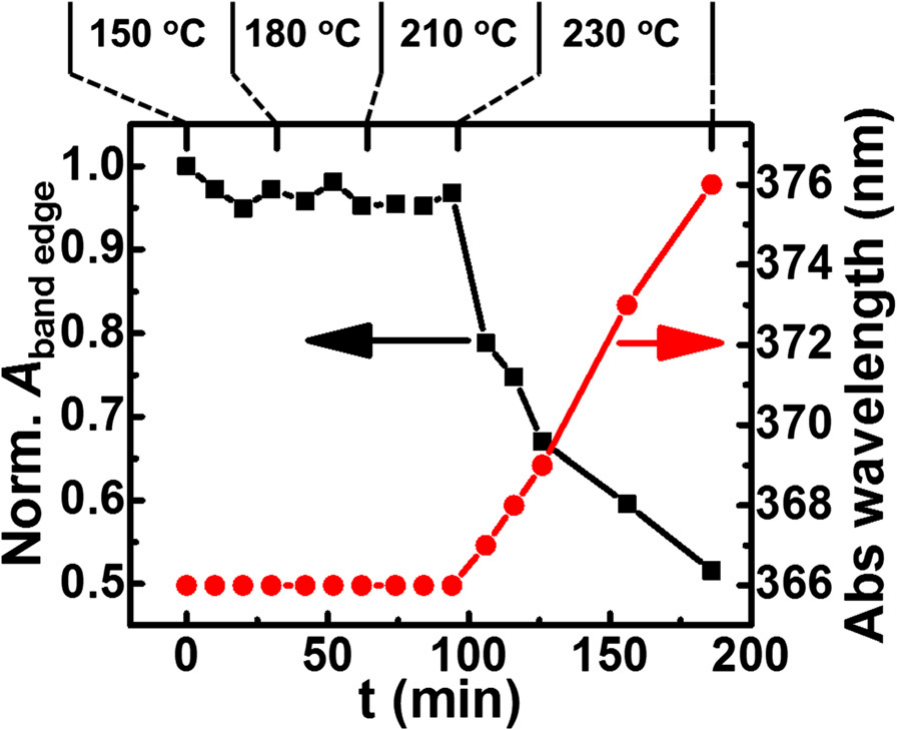
Band-edge PL integrated areas (normalized to set *t* = 0 min value as 1) and corresponding first absorption peak wavelengths of Mn-doped ZnS_x_Se_1−x_ NCs during low-temperature annealing. The NCs were grown at 260 °C for 30 min (without surface exchange/passivation) and then cooled down to 150 °C and annealed for 30 min; then, they were heated to 180 and 210 °C and annealed for 30 min, respectively; finally, they were heated to 230 °C and annealed for 90 min. All PL spectra are normalized to the absorbance at the excitation wavelength

Another possible cause of band-edge PL decrease is quenching PL by Mn surface adsorption on NCs that exhibit mainly band-edge PL during growth. However, annealing the reaction mixture at temperatures lower than when growth occurs but higher than the critical temperature for Mn surface adsorption (~150 °C) [32] reveals no band-edge PL change (data for *T* < 230 °C in Fig. 5). Even after surface exchange/passivation, with surface adsorbed Mn being replaced [46], the same trend of decreasing band-edge PL with growth time is also preserved. Therefore, Mn surface adsorption can be ruled out as a cause of the band-edge PL decrease. Further Mn incorporation into NCs with mainly band-edge PL can also be ruled out since no (isolated) Mn PL increase occurs along with band-edge PL decrease during extended growth (Fig. 2b).

**Fig. 6:**
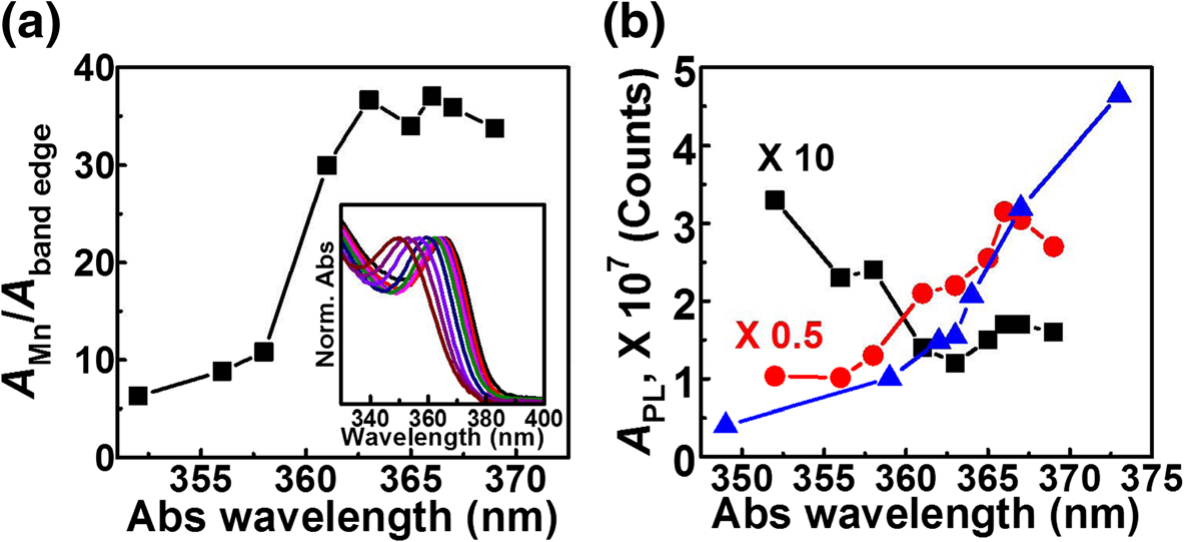
Size-selective precipitation results of Mn-doped ZnS_x_Se_1−x_ NCs with 15 min growth time (without surface exchange/passivation). **a** Ratios of the integrated area of Mn PL (including Mn-Mn PL) to that of band-edge PL of NCs from continuous size-selective precipitation (plotted versus the corresponding first absorption peak position). The *inset* shows the absorption spectra normalized to the first absorption peak. **b** Integrated areas of band-edge (*black squares*) and dopant (*red circles*) PL of the same NCs from continuous size-selective precipitation and band-edge PL of undoped NCs (*blue triangles*) shown in Additional file 1: Figure S2a (plotted versus the corresponding first absorption peak position). Band-edge PL integrated areas of undoped NCs are used as an indicator of the size-dependent band-edge PL QY. All PL spectra are normalized to the absorbance at the excitation wavelength

We then assert that this band-edge PL decrease despite the dopants being lost from the NCs is due to Ostwald ripening, i.e., *interparticle* ripening, reducing the number of undesirable NCs that exhibit mainly band-edge PL. Since larger particles grow at the expense of smaller particles in Ostwald ripening, if these undesirable NCs are the smaller NCs in the reaction mixture, one can expect them to dissolve away to allow growth of larger NCs that exhibit mainly Mn PL. Then, the knowledge of the distribution of NCs exhibiting mainly Mn PL vs. NCs exhibiting mainly band-edge PL with respect to size would be important and may be obtained through size selection. Absorption and PL spectra of the size-selected samples from a reaction mixture with 15 min growth (without the final ZnSt_2_ treatment) indeed verify that more undesirable NCs with mainly band-edge PL are the smaller ones. Figure 6a shows the absorption spectra of a series of samples from the size selection and how the corresponding ratio of Mn PL to band-edge PL (integrated area normalized to the absorbance at the excitation wavelength) varies. The corresponding series of PL spectra are shown in Additional file 1: Figure S5 and the Mn and band-edge PL dependence on size (or on the absorption peak position) is shown in Fig. 6b. The smaller NCs have higher band-edge PL, whereas the larger NCs have higher Mn PL. The decreasing band-edge PL with size (or with the absorption peak position) when the size dependence of PL QY exhibits the opposite trend as shown in Fig. 6b supports the idea that larger NCs are more likely to be doped and smaller NCs are less likely to be doped in a reaction ensemble. We note that a similar size-dependent PL based on size-selective precipitation has also been made on Mn doping of CdS NCs, although the lack of band-edge PL makes the comparison less straightforward [18]. This distribution of the degree of Mn doping with size for a given reaction batch may be explained by larger NCs being easier to dope [16, 20, 21, 31, 33, 34].

Given this distribution of Mn doping, Ostwald ripening reducing the number of smaller undesirable NCs can be expected. The decreasing band-edge PL that coincides with absorption red-shift in Fig. 5 (*T* = 230 °C) strongly supports this expectation. The correlation between the loss of smaller undesirable NCs with Ostwald ripening here is concurrent with Mn loss (Additional file 1: Figure S6), similar to the case for the growth at 260 °C. The net result is less absorption from the reduction in the number of NCs with mainly band-edge PL, which should enhance Mn PL QY. This benefit of Ostwald ripening works alongside dopant loss from heavily doped NCs which should also be important in increasing Mn PL QY. Interestingly, the former effect increases the average (ensemble) doping level, whereas the latter effect decreases the doping level. Hence, especially in the high dopant concentration regime as in our case, optimal doping situation can be achieved with an extended growth time (~90 min under current condition) due to dopant loss and Ostwald ripening working together synergistically.

### Other Host NC Compositions

**Fig. 7:**
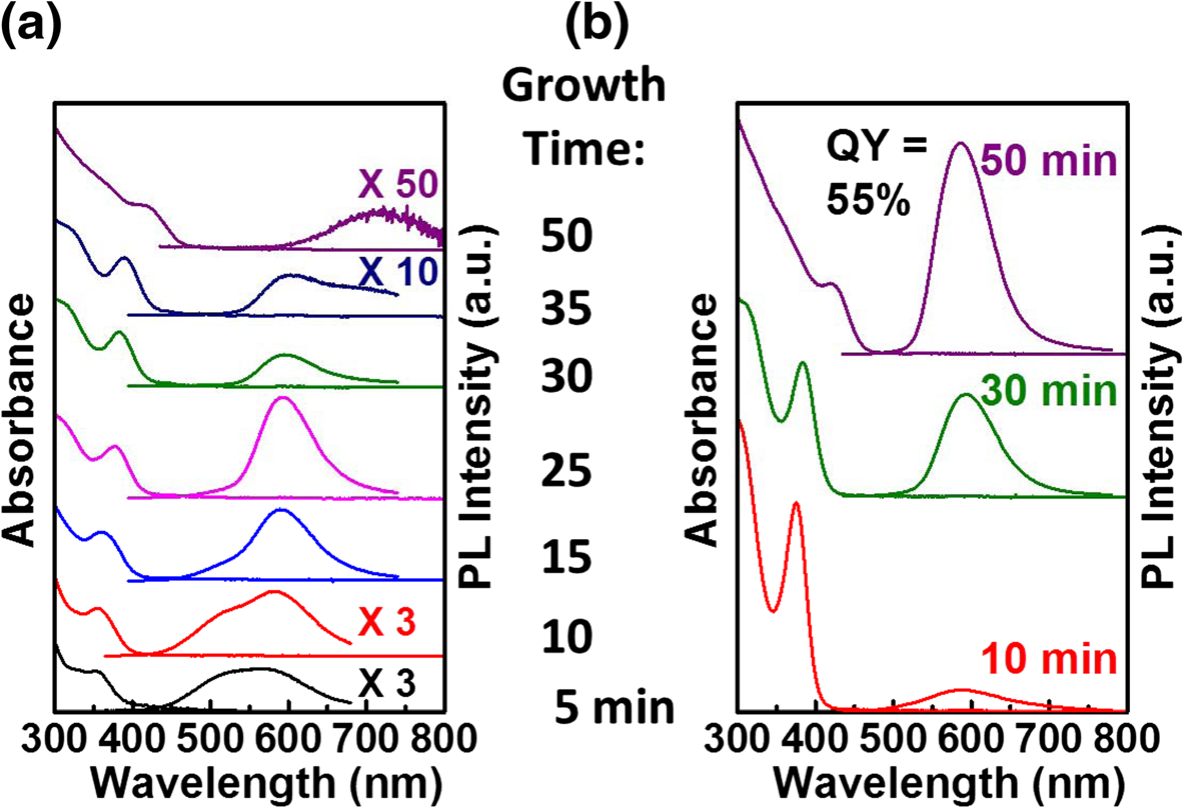
Absorption/PL spectra of Mn-doped CdS NCs at the indicated growth times before (**a**) and after (**b**) surface exchange/passivation. PL spectra in ( **a**) were collected at excitation wavelength of 350 nm for growth times of 5 and 10 min; 380 nm for 15, 25, 30, and 35 min; 420 nm for 50 min to accommodate absorption onset at shorter wavelengths for smaller NCs in earlier stages of growth. Spectra are offset for clarity. All PL spectra are normalized to the absorbance at the excitation wavelength

**Fig. 8:**
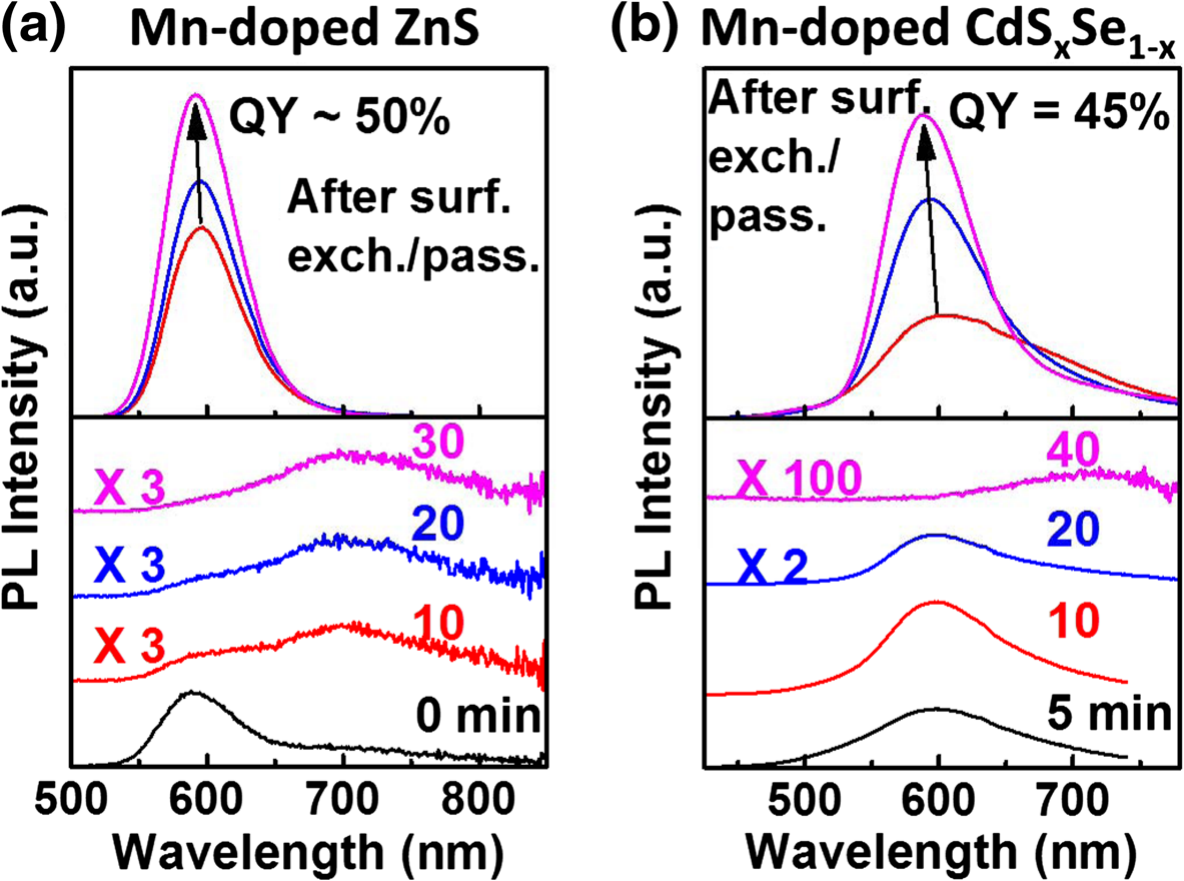
PL spectra of NCs at the indicated growth times before (*bottom*) and after (*top*) surface exchange/passivation for Mn-doped ZnS (**a**) and Mn-doped CdS_x_Se_1−x_ (**b**). *Bottom* spectra are offset for clarity. *Top* spectra (not offset) correspond to the last three growth times indicated for the bottom spectra. All PL spectra are normalized to the absorbance at the excitation wavelength

Optimizing the growth time of the heat-up synthesis to improve Mn doping can be extended to other host II-VI semiconductor NCs. Figure 7 shows the evolution of the absorption and PL spectra during growth (Fig. 7a) and after ZnSt_2_ surface exchange/passivation (Fig. 7b) for Mn doping of CdS. Although the lack of band-edge emission makes it less clear than the ZnS_x_Se_1−x_ case discussed above, the deep-trap emission (near 500 nm) from undesirable NCs shows similar trend of decreasing (although its size-dependent PL QY is clearly increasing, as shown in the undoped case in Additional file 1: Figure S7) prior to the maximum isolated Mn PL intensity being reached. The deep-trap emission can and does change in wavelength upon growth (and may overlap with the Mn PL), but the main Mn PL can be distinguished from it since the line widths are more than a factor of two different (deep-trap PL full-width-at-half-maximum ~120 nm based on results from undoped CdS NC growth shown in Additional file 1: Figure S7). Hence, such a PL change may also be explained by the reduction in the number of smaller undesirable NCs by Ostwald ripening. Upon ZnSt_2_ surface exchange/passivation, the Mn PL increases in intensity by more than 3 orders of magnitude, giving rise to a final Mn PL QY of 55 % for the case of 50 min growth. Again, similar to the ZnS_x_Se_1−x_ case, this maximum final PL QY case does not correspond to the growth time where the Mn PL shows the highest intensity prior to surface exchange/passivation but rather after extended growth (with significant Mn-Mn interaction PL) where we expect sufficient Mn loss to have enhanced PL QY of initially heavily doped NCs and Ostwald ripening to have reduced the number of undesirable NCs. Similar results can also be seen for Mn doping of ZnS and CdS_x_Se_1−x_ NCs (Fig. 8) where the high final Mn PL QY arises after extended growth when there is a significant Mn-Mn interaction PL prior to ZnSt_2_ surface exchange/passivation. The corresponding TEM images and EPR spectra for the final products of Mn-doped CdS, ZnS, and CdS_x_Se_1−x_ NCs are shown in Additional file 1: Figure S8.

## Conclusions

In conclusion, we have demonstrated how a simple heat-up method to synthesize Mn-doped II-VI NCs can benefit from optimized ripening/growth time where both dopant loss and Ostwald ripening can improve PL. High Mn concentrations achievable in this method lead to dopant loss, which has been considered unfavorable in many cases, actually increasing Mn PL QY. Interestingly, Ostwald ripening, another often unfavorable process, can also be beneficial because smaller NCs that are consumed for growth have higher undesirable band-edge PL. We have also shown that during ripening, there is a concurrent clustering of Mn dopants that occurs at or near the surface as evidenced by PL, EDS, and EPR measurements. Such clustering of Mn can be removed by additional cation precursors that can cause surface exchange/passivation. Hence, by a combination of optimized growth time and surface exchange/passivation, we have achieved high Mn PL QYs (45 to 55 %) in several different II-VI host NCs including alloy materials. Our better understanding of the doping and growth processes may facilitate future routes to further improve doped NCs through simple and scalable means.

## Additional Files

Additional file 1:
**Figure S1**. Spectral comparison between the reference dye C153 and all our Mn-doped NCs. **Figure S2**. Spectral evolution of undoped ZnS_**x**_ **Se**_**1−x**_ (including its band-edge PL change in comparison with the doped case) and **Figure S7** undoped CdS NCs. **Figure S3**. Growth time dependence of integrated PL areas of band-edge, isolated Mn, and Mn-Mn for other Mn precursor amounts. **Figure S4**. EDS spectra for composition analysis. **Figure S5**. Band-edge and Mn PL spectra for size-selective precipitation analysis. **Figure S6**. Mn PL spectra change during lower temperature annealing. **Figure S8**. TEM images and EPR spectra for Mn-doped ZnS, CdS, and CdS_**x**_ **Se**_**1−x**_ **NCs.**
