# Supplementary material for: Benefitting from Dopant Loss and Ostwald Ripening in Mn Doping of II-VI Semiconductor Nanocrystals
Source: Nanoscale Res Lett. 2015 Oct 28;10:423. doi: 10.1186/s11671-015-1123-9 (PMC4624686; doi:10.1186/s11671-015-1123-9)
Supplement: Additional file 1: — Figure S1. Spectral comparison between the reference dye C153 and all our Mn-doped NCs. Figure S2. Spectral evolution of undoped ZnSx Se 1−x (including its band-edge PL change in comparison with the doped case) and Figure S7 undoped CdS NCs. Figure S3. Growth time dependence of integrated PL areas of band-edge, isolated Mn, and Mn-Mn for other Mn precursor amounts. Figure S4. EDS spectra for composition analysis. Figure S5. Band-edge and Mn PL spectra for size-selective precipitation analysis. Figure S6. Mn PL spectra change during lower temperature annealing. Figure S8. TEM images and EPR spectra for Mn-doped ZnS, CdS, and CdSx Se 1−x NCs. [file 11671_2015_1123_MOESM1_ESM.pdf]

Supporting Information

# Benefitting from Dopant Loss and Ostwald Ripening in Mn Doping of II-VI Semiconductor Nanocrystals

*You Zhai and Moonsub Shim**

Department of Materials Science and Engineering, University of Illinois at Urbana-Champaign, Urbana, Illinois 61801, USA

*E-mail: mshim@illinois.edu. Tel: +1-217-333-7361.

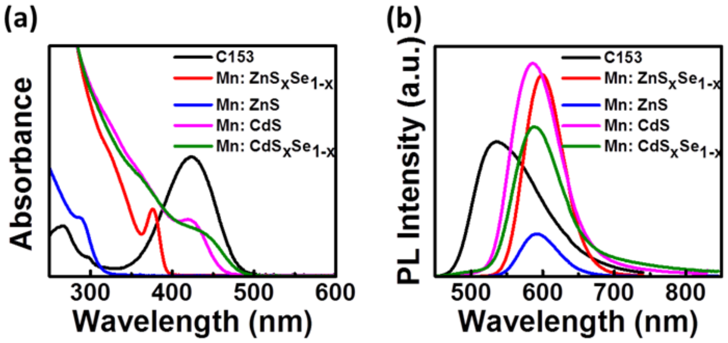


**Figure S1.** Absorption (a) and PL (b) spectra (not offset) of C153 and Mn-doped $ZnS_xSe_{1-x}$, ZnS, CdS, and $CdS_xSe_{1-x}$ NCs after surface exchange/passivation. All PL spectra are normalized to the absorbance at the excitation wavelength indicated in the main text. C153 spectrum shown here is for measuring the PL QY of Mn-doped $ZnS_xSe_{1-x}$ excited at 380 nm, but for other NCs separate spectra were collected with the same excitation wavelength and similar absorbance (< 0.1) at that wavelength as the NC being compared to (except for Mn-doped ZnS, where the excitation wavelength for C153 was still 380 nm).

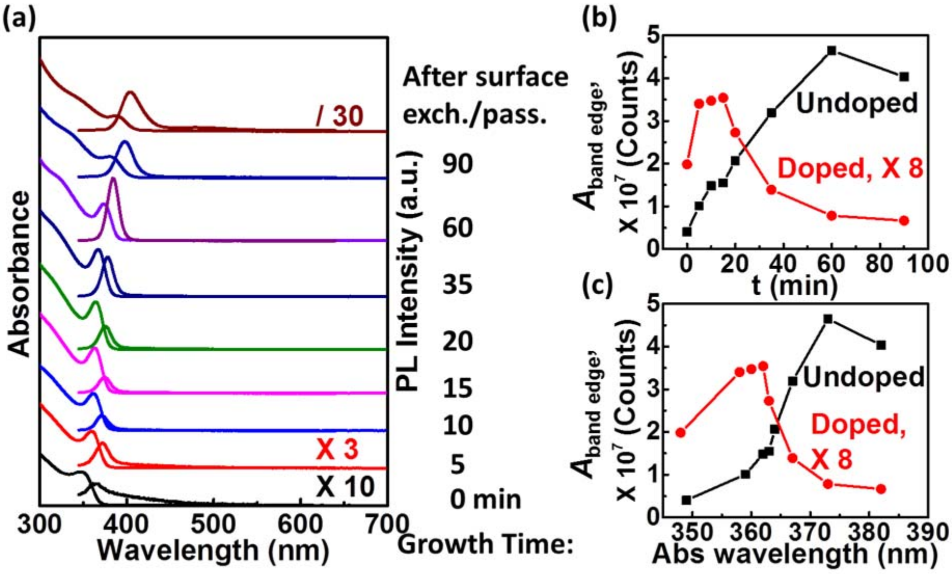


**Figure S2.** (a) Absorption/PL spectra of undoped $ZnS_xSe_{1-x}$ NCs at the indicated growth times before and after surface exchange/passivation. Spectra are offset for clarity. All PL spectra are normalized to the absorbance at the excitation wavelength. Dependence of band edge PL integrated area for undoped (shown in Figure S2a) and Mn-doped $ZnS_xSe_{1-x}$ NCs (shown in Figure 2a and 2b) on growth times (b) and their corresponding first absorption peak wavelengths (c). The undoped case is used as an indicator of the size dependent band edge PL QY.

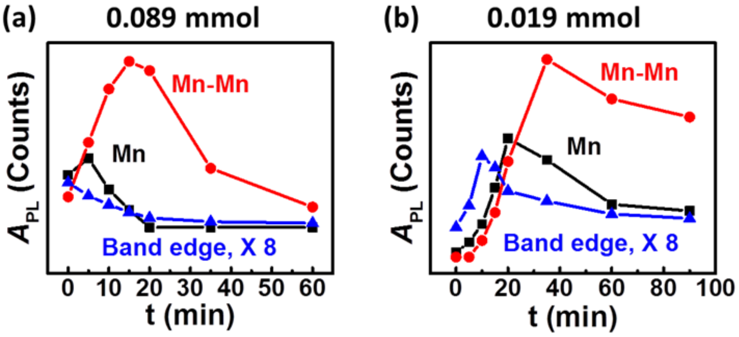


**Figure S3.** Growth time dependence of integrated PL areas of band edge, isolated Mn, and Mn-Mn interaction for Mn-doped $ZnS_xSe_{1-x}$ NCs before surface exchange/passivation for growth using 0.089 mmol (a) and 0.019 mmol (b) of $MnCl_2$ precursor. All PL spectra are normalized to the absorbance at the excitation wavelength.

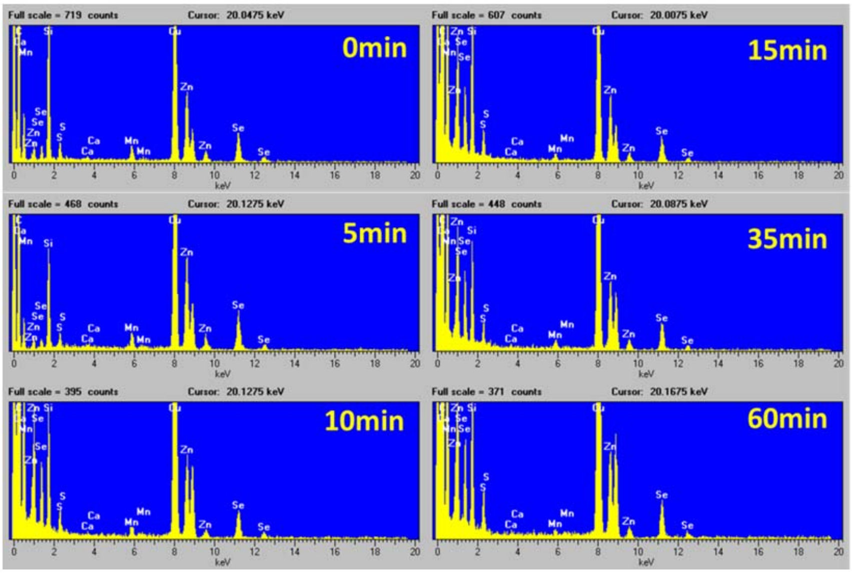


**Figure S4.** EDS spectra used for composition analysis shown in Figure 3a.

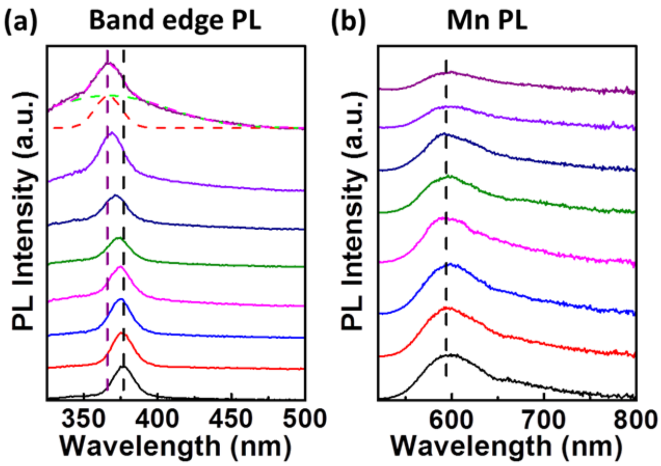


**Figure S5.** Band edge (a) and Mn (b) PL spectra used for size selective precipitation analysis shown in Figure 6. Vertical dashed lines indicate the initial and final peak positions. Dashed curves in (a) are the representative Gaussians used for peak fitting to remove the contribution of the broad PL from oleylamine residual. Spectra are offset for clarity. All PL spectra are normalized to the absorbance at the excitation wavelength.

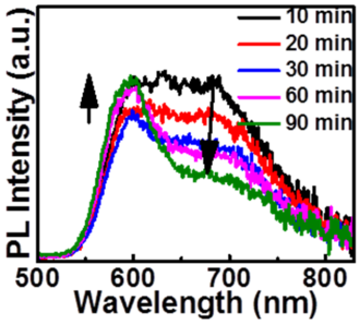


**Figure S6.** Evolution of PL spectrum at the indicated times during annealing the reaction mixture at 230 $^{o}$C (not offset). All PL spectra are normalized to the absorbance at the excitation wavelength. The reaction mixture has been grown at 260 $^{o}$C for 30 min before cooled down for low temperature annealing. At 230 $^{o}$C, the decrease of Mn-Mn PL and the recovery of isolated Mn PL indicate Mn loss.

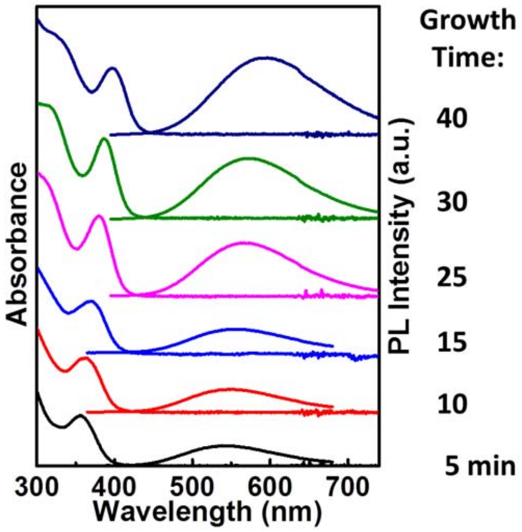


**Figure S7.** Absorption/PL spectra of undoped CdS NCs at the indicated growth times before surface exchange/passivation, demonstrating deep trap PL. PL spectra were collected with excitation wavelength of 350 nm for 5, 10, 15 min and 380 nm for 25, 30, 40 min to accommodate absorption onset at shorter wavelengths for smaller NCs in earlier stages of growth. Spectra are offset for clarity. All PL spectra are normalized to the absorbance at the excitation wavelength.

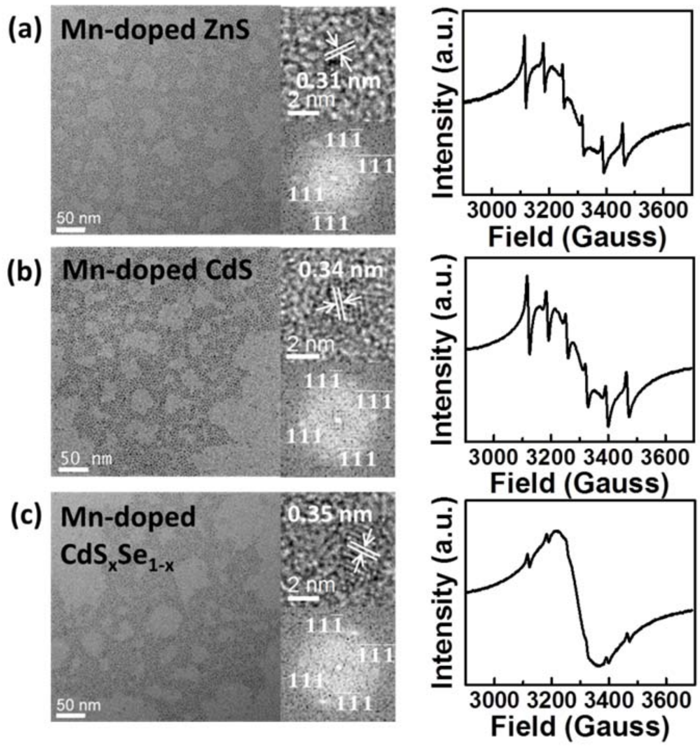


**Figure S8.** TEM images with HRTEM images shown in the top-right inset and corresponding FFT patterns shown in the bottom-right inset (left column) and EPR spectra (right column) of Mn-doped ZnS NCs (a), Mn-doped CdS NCs (b), and Mn-doped $CdS_xSe_{1-x}$ NCs (c) after surface exchange/passivation.
